# Interruptins Extracted from *Cyclosorus terminans* Protect Gut Pathologies Induced by High-Fat Diet in Rats

**DOI:** 10.3390/nu17081387

**Published:** 2025-04-20

**Authors:** Chanisa Thonusin, Kanokphong Suparan, Chanon Kunasol, Nopphakhun Lungruammit, Wichwara Nawara, Busarin Arunsak, Sasiwan Kerdphoo, Aphisek Kongkaew, Sujinda Songtrai, Hiranya Pintana, Chayodom Maneechote, Wasana Pratchayasakul, Sireewan Kaewsuwan, Nipon Chattipakorn, Siriporn C. Chattipakorn

**Affiliations:** 1 Cardiac Electrophysiology Unit, Department of Physiology, Faculty of Medicine, Chiang Mai University, Chiang Mai 50200, Thailand; chanisa.t@cmu.ac.th (C.T.); wasana.pratcha@cmu.ac.th (W.P.); nipon.chat@cmu.ac.th (N.C.); 2 Cardiac Electrophysiology Research and Training Center, Faculty of Medicine, Chiang Mai University, Chiang Mai 50200, Thailand; kanokphong.su@cmu.ac.th (K.S.); chanon.ku@cmu.ac.th (C.K.); wichwara.nawa@cmu.ac.th (W.N.); busarin.a@cmu.ac.th (B.A.); sasiwan.kerd@cmu.ac.th (S.K.); hiranya.pintana@cmu.ac.th (H.P.); chayodom.man@cmu.ac.th (C.M.); 3 Center of Excellence in Cardiac Electrophysiology Research, Chiang Mai University, Chiang Mai 50200, Thailand; 4 Faculty of Dentistry, Phayao University, Phayao 56000, Thailand; nopphakhun.lu@up.ac.th; 5 Research Administration Section, Faculty of Medicine, Chiang Mai University, Chiang Mai 50200, Thailand; aphisek.k@cmu.ac.th; 6 Faculty of Medical Technology, Rangsit University, Pathumthani 12000, Thailand; sujinda.s@rsu.ac.th; 7 Department of Pharmacognosy and Pharmaceutical Botany, Faculty of Pharmaceutical Sciences, Prince of Songkhla University, Songkhla 90110, Thailand; songsri.k@psu.ac.th; 8 Phytomedicine and Pharmaceutical Biotechnology Excellence Center, Faculty of Pharmaceutical Sciences, Prince of Songkhla University, Songkhla 90110, Thailand; 9 Department of Oral Biology and Diagnostic Sciences, Faculty of Dentistry, Chiang Mai University, Chiang Mai 50200, Thailand

**Keywords:** *Cyclosorus terminans*, gut, gut microbiota, interruptin, obesity

## Abstract

Background/Objectives: The fern “*Cyclosorus terminans*” (*C. terminans*) or “Maiden Fern” contains interruptin A and interruptin B. This plant could attenuate obesity, insulin resistance, and fatty liver in rats fed a high-fat/calorie diet. However, the benefits of *C. terminans* to the gut remain unknown. We investigated the protective effect of *C. terminans* extract against gut dysfunction in rats exposed to a high-fat/calorie diet. Methods: Male Wistar rats were assigned to receive either (1) a normal diet treated with vehicle, (2) a high-fat/calorie diet treated with vehicle, (3) a high-fat/calorie diet treated with 100 mg per kg per day (mg·kg^−1^·day^−1^) of *C. terminans* extract, or (4) a high-fat/calorie diet treated with 200 mg·kg^−1^·day^−1^ of *C. terminans* extract. The rats were euthanized after 12 weeks of treatment to enable feces and colon tissue collection. Results: Both 100 and 200 mg·kg^−1^·day^−1^ of *C. terminans* extract reduced body weight (−10.49%; *p* = 0.030 and −10.54%; *p* = 0.037, respectively) and ameliorated gut inflammation, gut barrier disruption, changes in short-chain fatty acid levels, and gut dysbiosis caused by high-fat/calorie diet. Conclusions: *C. terminans* extract attenuated an increase in body weight and exerted prophylactic effects against gut pathologies induced by high-fat/calorie diet.

## 1. Introduction

A strong association between obesity and gut microbiota has been widely established [1,2]. Previous studies have shown that obesity and obesity-related diseases are characterized by alterations in gut microbiota composition, known as “gut dysbiosis” [3,4,5]. In detail, it has been widely observed that obesity attributable to overconsumption of a high-fat/calorie diet led to increased inflammation of adipocytes, which resulted in the release of cytokines and chemokines into the circulation, known as “systemic inflammation” [6,7], which is a pathological process caused by systemic damage after a chronic inflammatory disease condition [8]. This consequently leads to gut inflammation and gut barrier disruption, finally contributing to gut dysbiosis and the changes in host-gut microbial cometabolite levels [9,10,11]. However, it was also found that gut dysbiosis played a critical role in the pathogenesis of obesity [12,13,14]. Indeed, gut dysbiosis-induced obesity was mediated by the changes in host-gut microbial cometabolite levels, resulting in a disruption of energy homeostasis, and consequently decreased energy expenditure [15,16]. Gut dysbiosis promoted gut inflammation and impaired gut barrier integrity and subsequently led to systemic inflammation [17,18,19,20,21,22]. Ultimately, it was found that the systemic inflammation is mechanistically linked to the pathophysiology of obesity [23]. According to all the mechanisms mentioned above, it is highly possible that any prophylactic interventions that help maintain the normal physiology of the gut can protect against high-fat/calorie diet-induced obesity and gut pathologies.

Currently, natural products are commonly applied as a therapeutic option for obesity-related diseases [24]. Our product of interest is “*Cyclosorus terminans* (J. Sm. ex Hook.) K.H. Shing” *(C. terminans)*, which is more well known as “Maiden Fern”. This plant is widely consumed in Thailand as a vegetable [25]. Interestingly, plants from the genus *Cyclosorus,* including *C. interruptus* and *C. acuminatus*, have been traditionally used. In fact, *C. interruptus* has been used for the treatment of burn, cough, and malaria [26,27], while *C. acuminatus* has been used for the treatment of inflammation, edema, and circulation stasis [28]. For these reasons, we expected that *C. terminans*, which is more commonly found in our country, Thailand, also exerts beneficial effects and can be used as a complementary therapeutic agent. The bioactive agents in *C. terminans* include interruptins A and B [29]. A prior in vitro study showed that *C. terminans* extract exerted an anti-diabetic property [29]. In addition, an in vivo study demonstrated that treatment with either 100 or 200 mg·kg^−1^·day^−1^ of *C. terminans* extract for two weeks reduced body weight, improved insulin sensitivity, increased HDL cholesterol level, and attenuated triglyceride accumulation in liver tissues of rats exposed to a high-fat/calorie diet [25]. Nonetheless, the effect of *C. terminans* as a protection against high-fat/calorie diet-induced gut dysfunction remains unknown.

After studying all the earlier research, we determined the prophylactic effects of *C. terminans* extract on high-fat/calorie diet-induced obesity and gut pathologies. In other words, we examined the connection between gut dysfunction and early intervention, which is important for the prevention of gastrointestinal disorders in obese condition. The indicators of gut function investigated in this study included gut inflammation, gut barrier integrity, host-gut microbial cometabolite levels, and the composition of gut microbiota. We hypothesized that *C. terminans* could protect against gut dysfunction in obese condition.

## 2. Materials and Methods

### 2.1. Study Protocol

**Figure 1:**
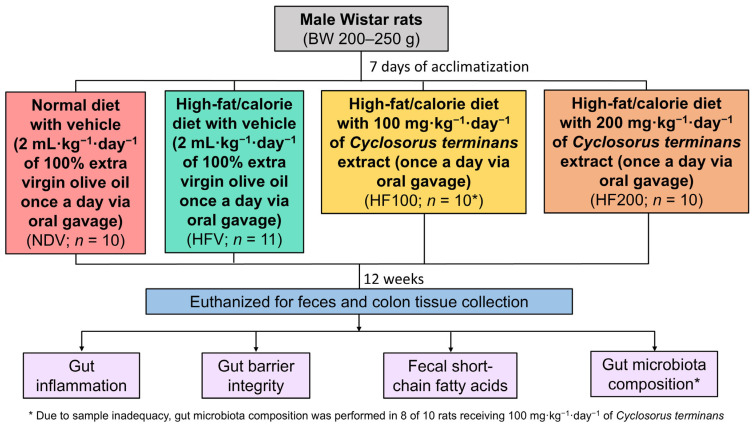
The study protocol. Rats were assigned to receive either (1) a normal diet with vehicle, (2) a high-fat/calorie diet with vehicle, (3) a high-fat/calorie diet with 100 mg·kg^−1^·day^−1^ of *C. terminans* extract, or (4) a high-fat/calorie diet with 200 mg·kg^−1^·day^−1^ of *C. terminans* extract. After 12 weeks of treatment, the rats were euthanized for molecular studies.

The protocol is shown in Figure 1. All experiments were approved by the Laboratory Animal Center, Chiang Mai University Animal Care and Use Committee, Chiang Mai University (approval number: 2565/RT-0016, approval date: 27 April 2022). Male Wistar rats with a body weight of 200–250 g were bought from Nomura Siam International, Bangkok, Thailand.

After 1 week of an acclimatization period, the rats were assigned to receive either (1) a normal diet treated with vehicle (NDV), (2) a high-fat/calorie diet treated with vehicle (HFV), (3) a high-fat/calorie diet treated with 100 mg·kg^−1^·day^−1^ of *C. terminans* extract (HF100), or (4) a high-fat/calorie diet treated with vehicle treated with 200 mg·kg^−1^·day^−1^ of *C. terminans* extract (HF200). The randomization was conducted using an animal randomization tool “https://acmeresearchlabs.in/animal-randomization-tool/ (accessed on 5 January 2022)” by ACME Research Solutions (New Delhi, India). The compositions of the diets were described in a previous study [30]. Since *C. terminans* extract can be completely dissolved in oil, the crude extract was dissolved in extra virgin olive oil before administering to the rats. The feeding volume of *C. terminans* extract was 2 mL·kg^−1^·day^−1^. Therefore, the vehicle used in this study was 2 mL·kg^−1^·day^−1^ of 100% extra virgin olive oil. Both vehicle and *C. terminans* extract were administered once a day via oral gavage. The doses of *C. terminans* extract were chosen based upon previous findings from our group, in which 100 and 200 mg· kg^−1^·day^−1^ of *C. terminans* attenuated obesity and fatty liver in obese rats [25]. The diets and treatment were continued for 12 weeks in each group.

At the end of treatment, the rats were sacrificed. Feces were collected for the quantification of short-chain fatty acid levels and the identification of gut microbiota composition. Colon tissues were obtained for the evaluation of gut inflammation and gut barrier integrity.

The blinding procedures were conducted during the experiments. In fact, people who treated the rats were not involved in the processes of euthanasia, molecular studies, and data analyses. In addition, the groups of rats were concealed from people who performed euthanasia, molecular studies, and data analyses.

### 2.2. Plant Extraction

Aerial parts of *C. terminans* were obtained. A voucher specimen (no. SKP 2080320001) was stored. The extraction method was detailed in a previous study [25]. The extract was evaluated for the amount of interruptins A (5.45% weight by weight) and B (1.41% weight by weight) by a high-performance liquid chromatography method [31]. From this method, the amount of a non-active ingredient—interruptin C (4.53% weight by weight)—was also determined.

### 2.3. Sample Size Calculation

According to a prior study showing that an extract from a plant *Emblica officinalis* could change gut microbial abundance and the related metabolites in obese mice [32], we used the acetic acid level to estimate the sample size. The previous findings yielded the effect size value of 0.67. Along with an alpha error probability of 0.05 and power of 0.80, the G*Power version 3.1.9.7 (Universität Kiel, Kiel, Germany) demonstrated the total sample size of 32 (8 per group).

### 2.4. The Expression of mRNA Analyses to Evaluate Gut Inflammation

The levels of mRNA expression of inflammatory-related genes including *TNF-α*, *IL-1β*, and *IL-6* were evaluated using a quantitative polymerase chain reaction, as detailed in a prior study [33]. All primer pairs are shown in Table S1.

### 2.5. Protein Expression Analyses to Evaluate Gut Barrier Integrity

Occludin and claudins play roles in the maintenance of epithelial barrier [34]. Hence, the expressions of occludin and claudin-5 proteins in colonic tissues were used as tight junction markers to determine gut barrier integrity. The protein expression analyses were performed using Western blot, which has been detailed in a previous study [35].

### 2.6. Quantification of Fecal Short-Chain Fatty Acids

The levels of fecal short-chain fatty acids, including acetic acid, propionic acid, butyric acid, and valeric acid were quantified using single quadrupole gas chromatography mass spectrometry, as detailed in a prior study [36]. This method has been specifically validated for the fecal samples used.

### 2.7. Identification of Gut Microbiota Composition

A genomic DNA isolation kit (QIAGEN, Hilden, Germany) was used to obtain bacterial DNA. The 250 mg of feces was extracted in accordance with the manufacturer’s instructions. Then, V3–V4 amplification and paired-end sequencing were performed and analyzed, as described in a prior study [36]. The taxonomy was annotated using a Naïve Bayes classifier implemented in Scikit-learn from the SILVA database version 138 (Leibniz Institute DSMZ-German Collection of Microorganisms and Cell Cultures GmbH, Braunschweig, Germany) [37,38].

### 2.8. Statistical Analyses

Alpha diversity and beta diversity between groups were compared using the Kruskal–Wallis test and permutational multivariate analysis of variance, respectively. The linear discriminant analysis effect size was performed to identify the taxa that were most likely to explain the differences in the gut microbiota composition between groups. The metrics for beta diversity included (1) Bray–Curtis, which examines the abundances of microbes that are shared between two samples, and the abundances of microbes found in each sample, (2) Jaccard, which determines the presence of microbes in one or both samples, (3) unweighted UniFrac, which defines the fraction of branch lengths between all gut microbes in two samples that show differences between the samples, and (4) weighted UniFrac, which resembles unweighted UniFrac but also takes the abundances of microbes into account [39]. All microbiome analyses were generated using R version 4.3.1 (the R Foundation for Statistical Computing Platform, Vienna, Austria). For the non-microbiome data, a parametric one-way analysis of variance followed by Fisher’s least significant difference method was conducted to detect the differences between groups using GraphPad Prism version 8 (GraphPad Software, San Diego, CA, USA). Spearman’s rank correlation coefficient was performed to examine the correlations between gut microbiota composition and other parameters using R version 4.3.1 (the R Foundation for Statistical Computing Platform, Vienna, Austria).

## 3. Results

### 3.1. C. terminans Extract Attenuated an Increase in Body Weight in Rats Exposed to High-Fat/Calorie Diet

**Figure 2:**
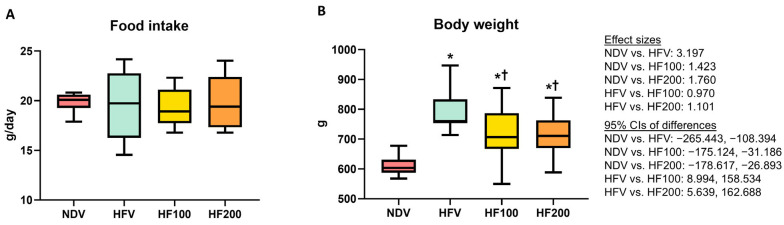
Food intake (**A**) and body weight (**B**). NDV: normal diet treated with vehicle (*n* = 10); HFV: high-fat/calorie diet treated with vehicle (*n* = 11); HF100: high-fat/calorie diet treated with 100 mg·kg^−1^·day^−1^ of *C. terminans* extract (*n* = 10); HF200: high-fat/calorie diet treated with 200 mg·kg^−1^·day^−1^ of *C. terminans* extract (*n* = 10); CIs: confidence intervals. * *p* < 0.05 when compared with NDV, ^†^ *p* < 0.05 when compared with HFV. The results showed that the HFV group exhibited increased body weight, when compared with the NDV group. The HF100 and HF200 groups had lower body weight than the HFV group, but the levels remained higher than that of the NDV group.

The side effects of the vehicle and *C. terminans* extract were not observed in our rats. The amount of food intake was no different between groups (Figure 2A). Following 12 weeks of high-fat/calorie diet consumption, the HFV group exhibited increased body weight, when compared to the NDV group (Figure 2B). The HF100 and HF200 groups had lower body weight than the HFV group, but the levels remained higher than that of the NDV group (Figure 2B). All of these findings indicated that *C. terminans* extract could ameliorate high-fat/calorie diet-induced increased body weight.

### 3.2. C. terminans Extract Exerted a Prophylactic Effect Against Gut Inflammation in Rats Exposed to High-Fat/Calorie Diet

**Figure 3:**
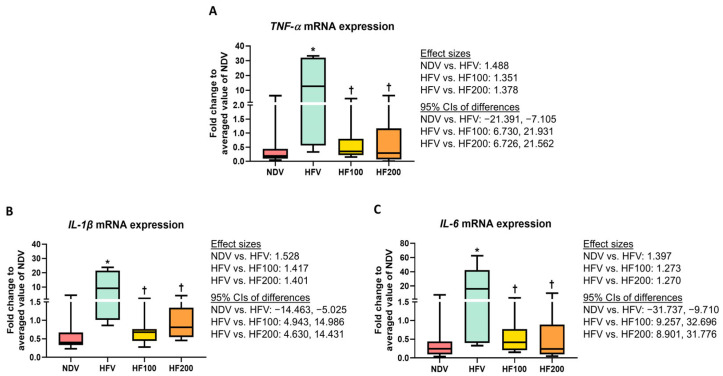
mRNA expression of inflammatory-related genes in colon tissues: *TNF-α* (**A**), *IL-1β* (**B**), and *IL-6* (**C**). NDV: normal diet treated with vehicle (*n* = 10); HFV: high-fat/calorie diet treated with vehicle (*n* = 11); HF100: high-fat/calorie diet treated with 100 mg·kg^−1^·day^−1^ of *C. terminans* extract (*n* = 10); HF200: high-fat/calorie diet treated with 200 mg·kg^−1^·day^−1^ of *C. terminans* extract (*n* = 10); CIs: confidence intervals. * *p* < 0.05 when compared with NDV, ^†^ *p* < 0.05 when compared with HFV. The results showed that *TNF-α*, *IL-1β*, and *IL-6* mRNA expressions were higher in colon tissues of the HFV group, indicating high-fat/calorie diet-induced gut inflammation. The HF100 and HF200 groups had lower *TNF-α*, *IL-1β*, and *IL-6* levels than those of the HFV group, and these levels did not differ from those of the NDV group.

The analysis of mRNA expression revealed that *TNF-α*, *IL-1β*, and *IL-6* levels were higher in the colons of the HFV group, when compared with those of the NDV group (Figure 3A–C), indicating high-fat/calorie diet-induced gut inflammation. The HF100 and HF200 groups had lower *TNF-α*, *IL-1β*, and *IL-6* levels than those of the HFV group, and these levels did not differ from those of the NDV group (Figure 3A–C). All of these results suggested that *C. terminans* extract exerted protection against gut inflammation caused by a high-fat/calorie diet.

### 3.3. C. terminans Extract Exerted a Prophylactic Effect Against Gut Barrier Disruption in Rats Exposed to High-Fat/Calorie Diet

**Figure 4:**
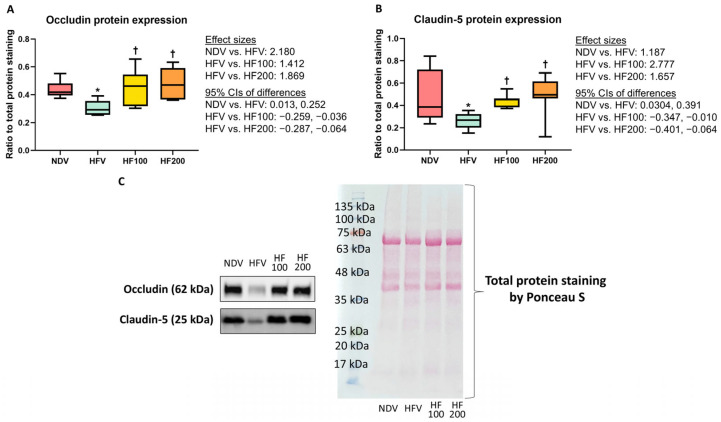
Expression of gut barrier proteins in colon tissues: occludin (**A**), claudin-5 (**B**), and representative Western blots (**C**). NDV: normal diet treated with vehicle (*n* = 10); HFV: high-fat/calorie diet treated with vehicle (*n* = 11); HF100: high-fat/calorie diet treated with 100 mg·kg^−1^·day^−1^ of *C. terminans* extract (*n* = 10); HF200: high-fat/calorie diet treated with 200 mg·kg^−1^·day^−1^ of *C. terminans* extract (*n* = 10); CIs: confidence intervals. * *p* < 0.05 when compared with NDV, ^†^ *p* < 0.05 when compared with HFV. The results showed that expression of occludin and claudin-5 proteins were lower in colon tissues of the HFV group, indicating high-fat/calorie diet-induced gut barrier disruption. The HF100 and HF200 groups displayed higher levels of these proteins in the colon, when compared to those of the HFV group, and these levels were no different from those of the NDV group.

Expressions of occludin and claudin-5 proteins were lower in the colon tissues of the HFV group than those of the NDV group (Figure 4A–C and Figure S1), indicating the high-fat/calorie diet-induced compromised gut barrier integrity. The HF100 and HF200 groups displayed higher levels of the expression of these proteins, when compared to those of the HFV group, and these levels were no different from those of the NDV group (Figure 4A–C and Figure S1). All of these findings suggested that *C. terminans* extract exerted a protection against gut barrier disruption caused by high-fat/calorie diet.

### 3.4. C. terminans Extract Exerted a Prophylactic Effect Against the Alterations of Fecal Short-Chain Fatty Acid Levels in Rats Exposed to High-Fat/Calorie Diet

**Figure 5:**
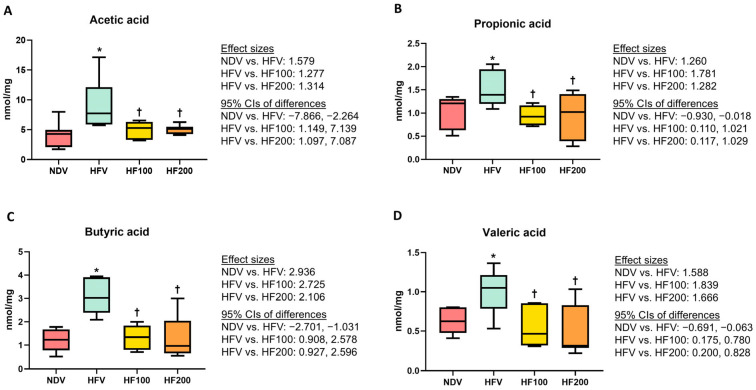
Fecal short-chain fatty acid levels: acetic acid (**A**), propionic acid (**B**), butyric acid (**C**), and valeric acid (**D**). NDV: normal diet treated with vehicle (*n* = 10); HFV: high-fat/calorie diet treated with vehicle (*n* = 11); HF100: high-fat/calorie diet treated with 100 mg·kg^−1^·day^−1^ of *C. terminans* extract (*n* = 10); HF200: high-fat/calorie diet treated with 200 mg·kg^−1^·day^−1^ of *C. terminans* extract (*n* = 10); CIs: confidence intervals. * *p* < 0.05 when compared with NDV, ^†^ *p* < 0.05 when compared with HFV. The HFV group exhibited greater levels of fecal short-chain fatty acids. The HF100 and HF200 groups had lower levels of fecal short-chain fatty acids than those of the HFV group, and these levels did not differ from those of the NDV group.

It was demonstrated that the HFV group exhibited greater levels of acetic acid, propionic acid, butyric acid, and valeric acid, when compared with those in the NDV group (Figure 5A–D). The HF100 and HF200 groups had lower levels of all four fecal short-chain fatty acids than those of the HFV group, and these levels did not differ from those of the NDV group (Figure 5A–D). All of these results suggested that *C. terminans* extract exerted protection against the alterations of host-gut microbial cometabolite levels in rats exposed to high-fat/calorie diet.

### 3.5. C. terminans Extract Exerted a Prophylactic Effect Against the Alteration of Alpha Diversity in Rats Exposed to High-Fat/Calorie Diet

**Figure 6:**
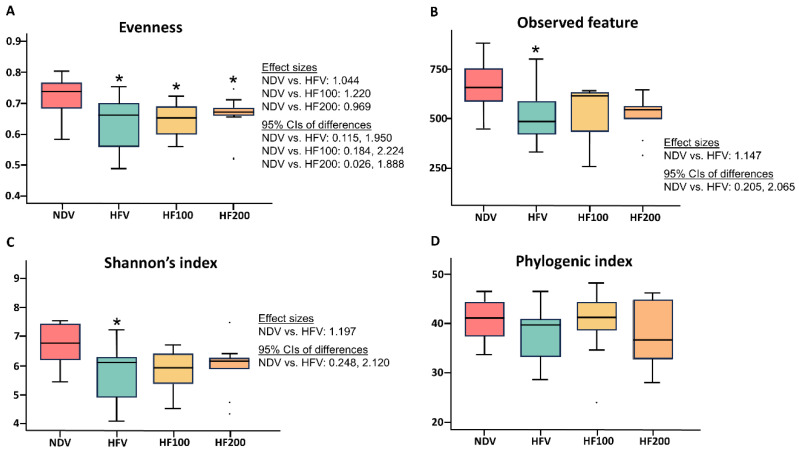
Alpha diversity: evenness (**A**), observed feature (**B**), Shannon’s index (**C**), and phylogenic index (**D**). NDV: normal diet treated with vehicle (*n* = 10); HFV: high-fat/calorie diet treated with vehicle (*n* = 11); HF100: high-fat/calorie diet treated with 100 mg·kg^−1^·day^−1^ of *C. terminans* extract (*n* = 10); HF200: high-fat/calorie diet treated with 200 mg·kg^−1^·day^−1^ of *C. terminans* extract (*n* = 10); CIs: confidence intervals. * *p* < 0.05 when compared with NDV. Compared to those of the NDV group, the HFV group exhibited decreased evenness, observed features, and Shannon’s index of alpha diversity, whereas phylogenic index was no different between groups. Both 100 mg·kg^−1^·day^−1^ and 200 mg·kg^−1^·day^−1^ of *C. terminans* extract could attenuate the reduction in observed features and Shannon’s index of alpha diversity. Nonetheless, neither 100 mg·kg^−1^·day^−1^ nor 200 mg·kg^−1^·day^−1^ of *C. terminans* extract improved evenness of alpha diversity.

Four metrics of alpha diversity were determined. These included (1) evenness, which refers to how similar the abundances of different species are in the community, (2) observed feature, which represents community richness, (3) Shannon’s index, which measures the diversity of species in a community, and (4) phylogenic index, which also identifies the diversity of species, but it is specific to the phylogenetic tree [40]. Compared with those of the NDV group, the HFV group exhibited decreased evenness, observed feature, as well as Shannon’s index of alpha diversity (Figure 6A–C), whereas the phylogenic index was no different between groups (Figure 6D). Both 100 mg·kg^−1^·day^−1^ and 200 mg·kg^−1^·day^−1^ of *C. terminans* extract could attenuate the reduction in observed features and Shannon’s index of alpha diversity in rats exposed to high-fat/calorie diet (Figure 6B,C). Nonetheless, neither 100 mg·kg^−1^·day^−1^ nor 200 mg·kg^−1^·day^−1^ of *C. terminans* extract improved the evenness of alpha diversity in rats exposed to a high-fat/calorie diet (Figure 6A).

### 3.6. C. terminans Extract Did Not Improve Beta Diversity in Rats Fed High-Fat/Calorie Diet

**Figure 7:**
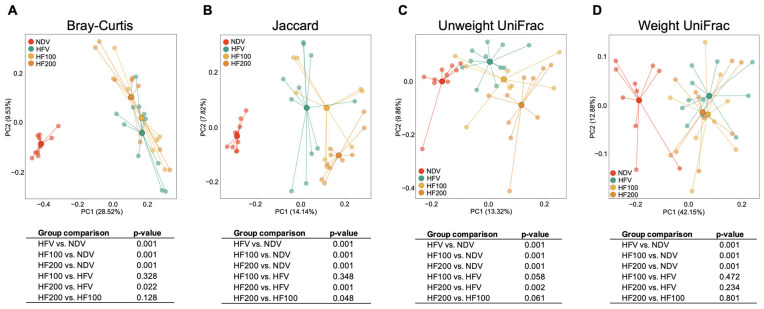
Beta diversity: Bray–Curtis (**A**), Jaccard (**B**), unweighted UniFrac (**C**), and weighted UniFrac (**D**). NDV: normal diet treated with vehicle (*n* = 10); HFV: high-fat/calorie diet treated with vehicle (*n* = 11); HF100: high-fat/calorie diet treated with 100 mg·kg^−1^·day^−1^ of *C. terminans* extract (*n* = 8); HF200: high-fat/calorie diet treated with 200 mg·kg^−1^·day^−1^ of *C. terminans* extract (*n* = 10). Each dot represents the gut microbial composition of an individual rat, with lines connecting samples to their respective group centroids. The results showed that the beta diversity metrics differed between the NDV group and the HFV group, indicating high-fat/calorie diet-induced change in beta diversity. Treatment with 100 mg·kg^−1^·day^−1^ of *C. terminans* extract could not alleviate the change in beta diversity. Interestingly, the differences in Bray–Curtis, Jaccard, and unweighted UniFrac were revealed between the HFV group and the HF200 group. However, all these remained different between the NDV group and the HF200 group.

Beta diversity is illustrated by principal component analysis, as shown in Figure 7A–D. Each dot represents the gut microbial composition of an individual rat, with lines connecting samples to their respective group centroids (Figure 7A–D). The variance values ranged from 7.82% to 42.15% (Figure 7A–D). Clustering distances between the NDV group and the other groups indicated that the NDV group exerted obvious dissimilarities (Figure 7A–D). Specifically, Bray–Curtis, Jaccard, unweighted UniFrac, and weighted UniFrac differed between the NDV group and the HFV group (Figure 7A–D), indicating high-fat/calorie diet-induced change in beta diversity. However, 100 mg·kg^−1^·day^−1^ of *C. terminans* extract could not alleviate the change in beta diversity induced by a high-fat/calorie diet, as indicated by the overlapping clusters and no difference in any beta diversity metrics between the HFV group and the HF100 group (Figure 7A–D). Interestingly, the clustering distances of Bray–Curtis, Jaccard, and unweighted UniFrac between the HFV group and the HF200 group were revealed, which reached statistical significance (Figure 7A–C). Nevertheless, all these remained different between the NDV group and the HF200 group (Figure 7A–D). All of these results suggested that *C. terminans* extract did not improve beta diversity in rats exposed to a high-fat/calorie diet. Moreover, 200 mg·kg^−1^·day^−1^ of *C. terminans* extract caused the alteration in beta diversity in rats exposed to high-fat/calorie diet.

### 3.7. Differential Abundance of Gut Microbiota Composition

**Figure 8:**
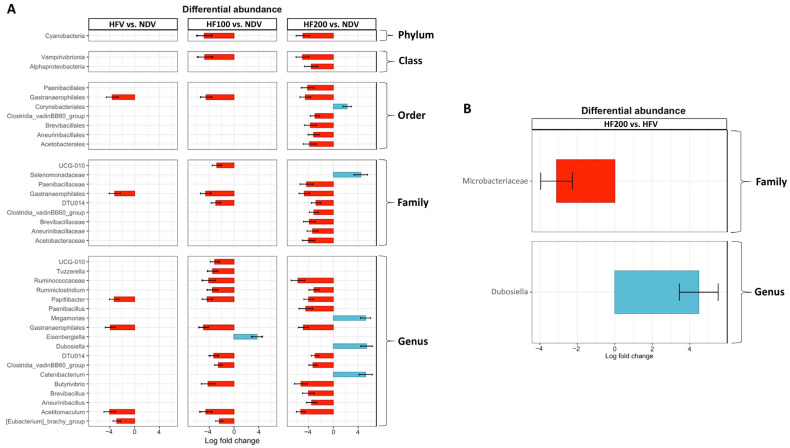
Differential gut microbial abundance that showed a significant difference from NDV (**A**) and HFV (**B**). NDV: normal diet treated with vehicle (*n* = 10); HFV: high-fat/calorie diet treated with vehicle (*n* = 11); HF100: high-fat/calorie diet treated with 100 mg·kg^−1^·day^−1^ of *C. terminans* extract (*n* = 8); HF200: high-fat/calorie diet treated with 200 mg·kg^−1^·day^−1^ of *C. terminans* extract (*n* = 10). Data are shown as mean ± standard error of the mean. The HFV group exhibited increases in the order Gastranaerophilales, family Gastranaerophilales, genus *Gastranaerophilales*, genus *Papillibacter*, and genus *Acetitomaculum*, when compared to the NDV group. In the HF100 and HF200 groups, these changes remained present, along with the decreases in several gut microbiomes, specifically genus *Tuzzerella*, genus *Ruminococcaceae*, genus *Ruminiclostridium*, genus *Paenibacillus*, genus *Butyrivibrio*, genus *Brevibacillus*, genus *Aneurinibacillus*, and genus *Acetitomaculum*. On the other hand, increases in genus *Megamonas*, genus *Dubosiella*, genus *Catenibacterium*, and genus *Eisenbergiella* were found in the HF 100 and HF200 groups, when compared to those of the NDV group. When compared with the HFV group, the HF200 group exhibited a reduction in family Microbacteriaceae, along with an increase in genus *Dubosiella*.

In addition to beta diversity, we evaluated differential gut microbial abundance. The results are shown in Figures S2 and S3. Focusing on the gut microbiomes that were significantly different between groups, we found that the HFV group exhibited increases in the order Gastranaerophilales, family Gastranaerophilales, genus *Gastranaerophilales*, genus *Papillibacter*, and genus *Acetitomaculum*, when compared to those of the NDV group (Figure 7A). In the HF 100 and HF 200 groups, these changes remained present, along with the decreases in several gut microbiomes, specifically genus *Tuzzerella*, genus *Ruminococcaceae*, genus *Ruminiclostridium*, genus *Paenibacillus*, genus *Butyrivibrio*, genus *Brevibacillus*, genus *Aneurinibacillus*, and genus *Acetitomaculum* (details are shown in Figure 8A). On the other hand, increases in genus *Megamonas*, genus *Dubosiella*, genus *Catenibacterium*, and genus *Eisenbergiella* were found in the HF100 and HF200 groups, when compared to those of the NDV group (details are shown in Figure 8A).

When compared to the HFV group, the HF200 group revealed a reduction in family Microbacteriaceae, along with an increase in genus *Dubosiella* (Figure 8B). Nonetheless, there was no significant difference in gut microbial abundance between the HFV group and the HF100 group, as well as between the HF100 group and the HF200 group.

### 3.8. The Abundance of Genus Dubosiella Was Positively Correlated with Claudin-5 Protein Expression in Colon Tissues

**Figure 9:**
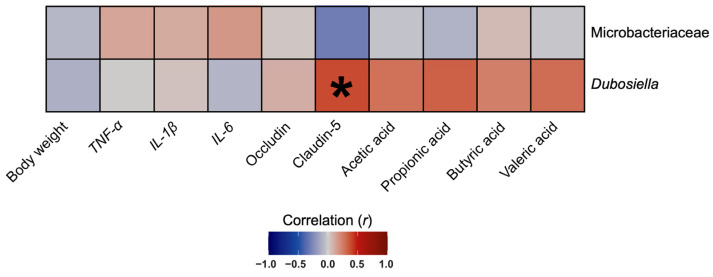
Spearman’s rank correlation between family Microbacteriaceae and genus *Dubosiella* versus body weight and other gut-related parameters. The results were calculated from all four groups of rats (*n* = 39). * *p* < 0.05. It was shown that the amount of genus *Dubosiella* was positively correlated with claudin-5 protein expression in colon tissues.

To identify whether the effects of the *C. terminans* extract on gut microbiota composition were related to body weight and other gut-related outcomes or not, we determined the correlations between gut microbial abundance versus body weight, inflammatory markers, gut barrier integrity markers, and short-chain fatty acid levels. We focused on the abundance of family Microbacteriaceae and genus *Dubosiella*: the only two microbiomes that were changed in our rats exposed to high-fat/calorie diet after treatment with *C. terminans* extract. We found a significantly positive correlation between genus *Dubosiella* and claudin-5 protein expression in the colon tissues of our rats (Figure 9). This result suggested that the improvement in claudin-5 protein expression induced by *C. terminans* extract was associated with an increase in genus *Dubosiella*.

## 4. Discussion

We examined the prophylactic effects of *C. terminans* extract against high-fat/calorie diet-induced obesity and gut dysfunction. Our vehicle-treated rats were exposed to high-fat/calorie diet to induce obesity, and we expected that obesity subsequently caused gut dysfunction. The results showed that we successfully developed a rat model of obesity with gut dysfunction, as indicated by increased body weight, increased gut inflammation, gut barrier disruption, altered short-chain fatty acid levels, and gut dysbiosis in the HFV group, when compared to those of the NDV group.

The result showed that the early treatment with *C. terminans* extract ameliorated an increase in body weight in rats exposed to a high-fat/calorie diet. These effects were likely to be mediated by the benefits of interruptins in promoting brown adipogenic differentiation, which were revealed in a prior in vitro study [29]. Mechanistically, brown adipogenic differentiation promotes lipolysis and increases total energy expenditure [41], all of which prevent the development of obesity [42]. Interestingly, we observed that these effects of *C. terminans* extract were comparable between the doses of 100 mg·kg^−1^·day^−1^ and 200 mg^−1^ kg^−1^ day^−1^. These findings are inconsistent with a prior study as they show that 2 weeks of treatment with 200 mg·kg^−1^·day^−1^ of *C. terminans* extract was superior to that of 100 mg·kg^−1^·day^−1^ in attenuating increased body weight in rats previously exposed to 12 weeks of high-fat/calorie diet [25]. All of these results highlight the greater benefits of early treatment.

Previous understanding regarding the pathophysiology of obesity-induced gut dysbiosis [9,10,11] has indicated that this prophylactic effect of *C. terminans* extract in our rats exposed to a high-fat/calorie diet began with protection against gut inflammation, resulting in a protection against gut barrier disruption, ultimately leading to a protection against the changes in alpha diversity. Nevertheless, our results failed to explain whether the protective effect of *C. terminans* extract on the gut was the direct effect on the gut itself or it was mediated by the benefit of *C. terminans* extract in protecting against increased body weight. Since gut dysbiosis also played a pivotal role in the development of obesity [12,13,14], our findings could not explain whether the prophylactic effect of *C. terminans* extract against obesity was mediated by the protective effect of *C. terminans* extract against the change in alpha diversity. Indeed, the mechanistic link between *C. terminans* extract, gut dysbiosis, and obesity could not be holistically determined in this study. This may limit the use of *C. terminans* extract in future clinical practice. For these reasons, future experiments in an animal model of gut dysbiosis-induced obesity or a different time-point study into high-fat/calorie diet consumption with *C. terminans* extract is required.

In addition to the impact of gut microbiomes on energy homeostasis and the pathophysiology of obesity [12,13,14], a healthy gut exerts a pivotal role in maintaining the physiological function of other organ systems [43,44]. These include the nervous system [45], cardiovascular system [46], and skeletal system [47]. For this reason, we speculated that *C. terminans* extract also exerts protection against neurological disorders, cardiovascular diseases, and bone remodeling induced by obesity. To verify these benefits of *C. terminans* extract, future studies evaluating the effect of this extract on the brain, heart, and bone are needed.

Although *C. terminans* extract could not improve beta diversity in our rats exposed to high-fat/calorie diet, we found that the abundances of some gut microbiomes were significantly altered only in rats exposed to high-fat/calorie diet treated with this extract. For example, a decrease in genus *Tuzzerella*: a microbiome that is associated with immune dysfunction [48], the reduction in opportunistic pathogens: genus *Paenibacillus* and genus *Brevibacillus* [49,50], an increase in genus *Dubosiella* that is a probiotic [51], and an increase in genus *Catenibacterium* that is associated with the lower cardiovascular disease risk [52]. All of these findings suggested that *C. terminans* extract could promote a more favorable gut microbiome profile.

A positive correlation between genus *Dubosiella* and claudin-5 protein expression in colon tissues was observed in our rats, suggesting an association between this gut microbiome and gut barrier integrity. This was consistent with a prior study demonstrating that treatment with *Dubosiella newyorkensis* attenuated gut barrier injury in mice with colitis [53]. All of these have highlighted the benefit of *Dubosiella* in improving gut barrier integrity, both in obesity and colitis.

This study has some limitations. The sample size for gut microbiota analysis across groups was varied, which might affect the validity of our findings. Importantly, our results could not clarify the mechanistic insight into the relationship between *C. terminans* extract, obesity, and gut dysbiosis. Therefore, a study with an equal sample size across groups and a study in a model of gut dysbiosis-induced obesity or a different time-point study into high-fat/calorie diet consumption with *C. terminans* extract are needed in the future.

## 5. Conclusions

The findings of this study revealed the potential for *C. terminans* extract to alleviate the increase in body weight and protect against gut dysfunction induced by high-fat/calorie diet. Nonetheless, further long-term studies identifying the safety of the use of *C. terminans* extract and further studies comparing the efficacy between *C. terminans* extract and other paradigms of gut microbiome modulation in animals are warranted and necessary, prior to the use of *C. terminans* extract in clinical trials.

## Data Availability

The datasets generated and/or analyzed during the current study have been deposited in the National Center for Biotechnology Information (NCBI) Sequence Read Archive (SRA) under the BioProject accession number PRJNA1249221, with individual sample accessions ranging from SRA:47883419 to SRA:47883457.

## Supplementary Materials

The following supporting information can be downloaded at: https://www.mdpi.com/article/10.3390/nu17081387/s1, Table S1: primer pairs used in this study; Figure S1: original Western blots for gut barrier-related proteins; Figure S2: differential abundance of gut microbiota composition (family level); Figure S3: differential abundance of gut microbiota composition (genus level).

## Abbreviations

The following abbreviations are used in this manuscript:

*C. terminans*	*Cyclosorus terminans*
DNA	deoxyribonucleic acid
HDL	high-density lipoprotein
HFV	high-fat/calorie diet treated with vehicle
HF100	high-fat/calorie diet treated with 100 mg·kg^−1^·day^−1^ of *C. terminans* extract
HF200	high-fat/calorie diet treated with 200 mg·kg^−1^·day^−1^ of *C. terminans* extract
IL-1β	interleukin-1beta
IL-6	interleukin-6
mg·kg^−1^·day^−1^	milligram per kilogram per day
mRNA	messenger ribonucleic acid
NCBI	National Center for Biotechnology Information
NDV	normal diet treated with vehicle
SRA	Sequence Read Archive
TNF-α	tumor necrosis factor-alpha
