# Supplementary material for: Interruptins Extracted from Cyclosorus terminans Protect Gut Pathologies Induced by High-Fat Diet in Rats
Source: Nutrients. 2025 Apr 20;17(8):1387. doi: 10.3390/nu17081387 (PMC12030309; doi:10.3390/nu17081387)
Supplement: Supplementary file 1 [file nutrients-17-01387-s001.zip › nutrients-3571845-supplementary.pdf]

Table S1. *Primer pairs used in this study*

**Primers for *Rattus norvegicus***	**Sequences**
TNF-α	5’-ACTCCCAGAAAAGCAAGCAA-3’
	3’-CGAGCAGGAATGAGAAGAGG-5’
IL-1β	5’-CACCTCTCAAGCAGAGCACAG-3’
	3’-GGGTTCCATGGTGAAGTCAAC-5’
IL-6	5’-TCCTACCCCAACTTCCAATGCTC-3’
	3’-TTGGATGGTCTTGGTCCTTAGCC-5’
β-actin	5’-GGAGATTACTGCCCTGGCTCCTA-3’
	3’-GACTCATCGTACTCCTGCTTGCTG-5’

Figure S1. *Original Western blots for gut barrier-related proteins*

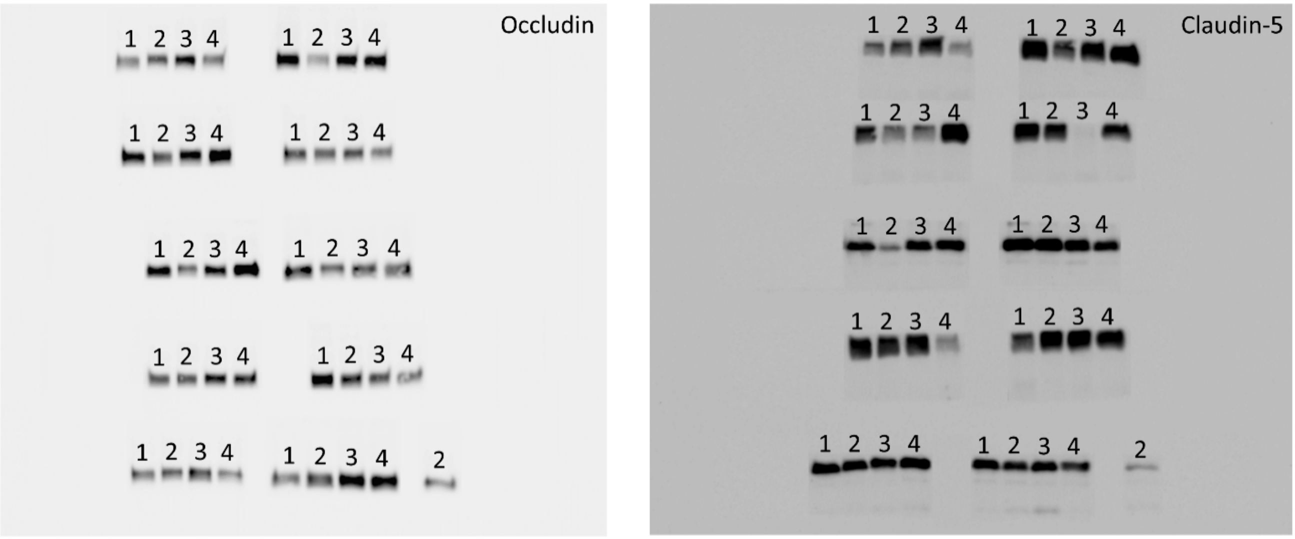

1 = NDV: normal diet treated with vehicle

2 = HFV: high-fat/calorie diet treated with vehicle

3 = HF100: high-fat/calorie diet treated with 100 mg·kg^{-1}·day^{-1} of *C. terminans* extract

4 = HF200: high-fat/calorie diet treated with 200 mg·kg^{-1}·day^{-1} of *C. terminans* extract

Figure S2. *Differential abundance of gut microbiota composition (family level)*

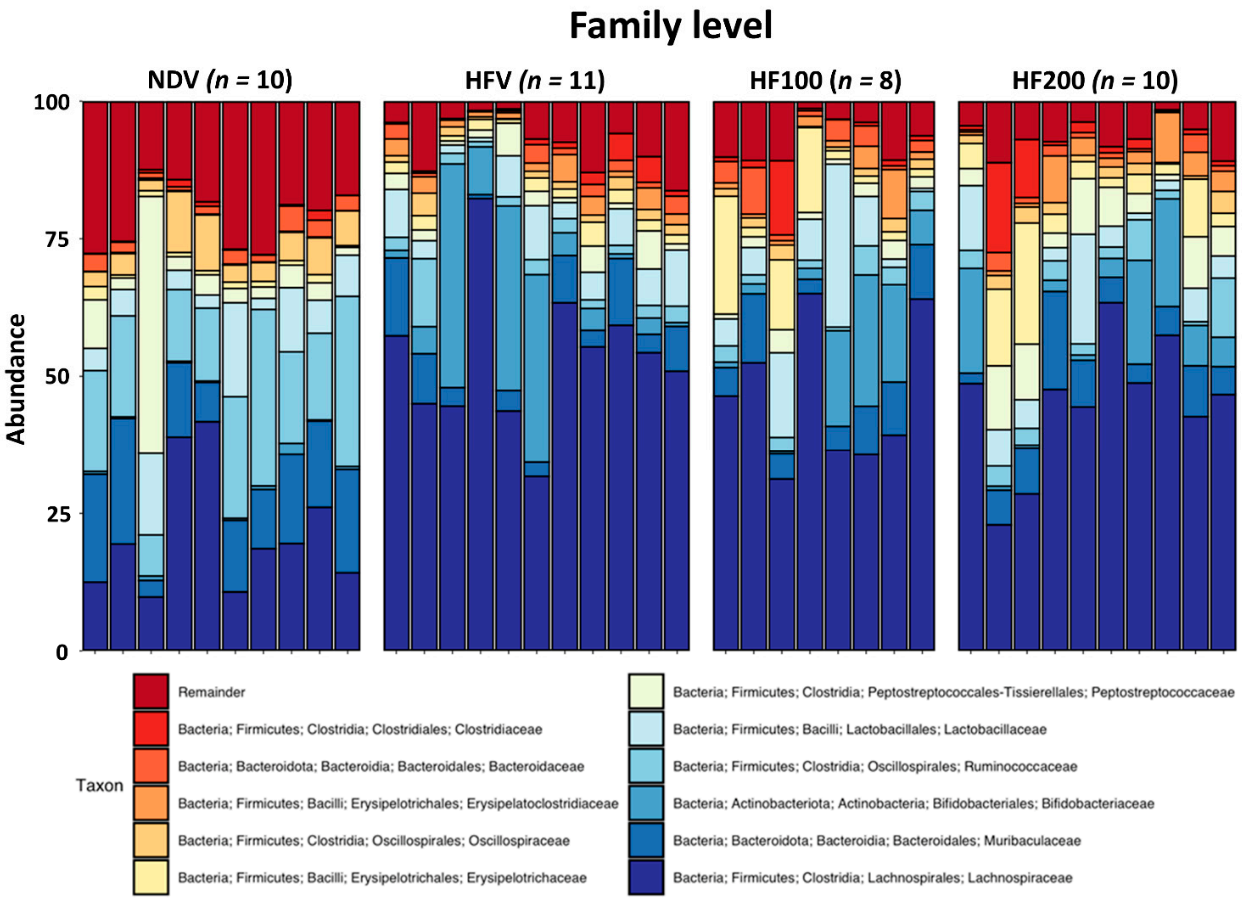

NDV: normal diet treated with vehicle

HFV: high-fat/calorie diet treated with vehicle

HF100: high-fat/calorie diet treated with 100 $mg \cdot kg^{-1} \cdot day^{-1}$ of *C. terminans* extract

HF200: high-fat/calorie diet treated with 200 $mg \cdot kg^{-1} \cdot day^{-1}$ of *C. terminans* extract

Figure S3. *Differential abundance of gut microbiota composition (genus level)*

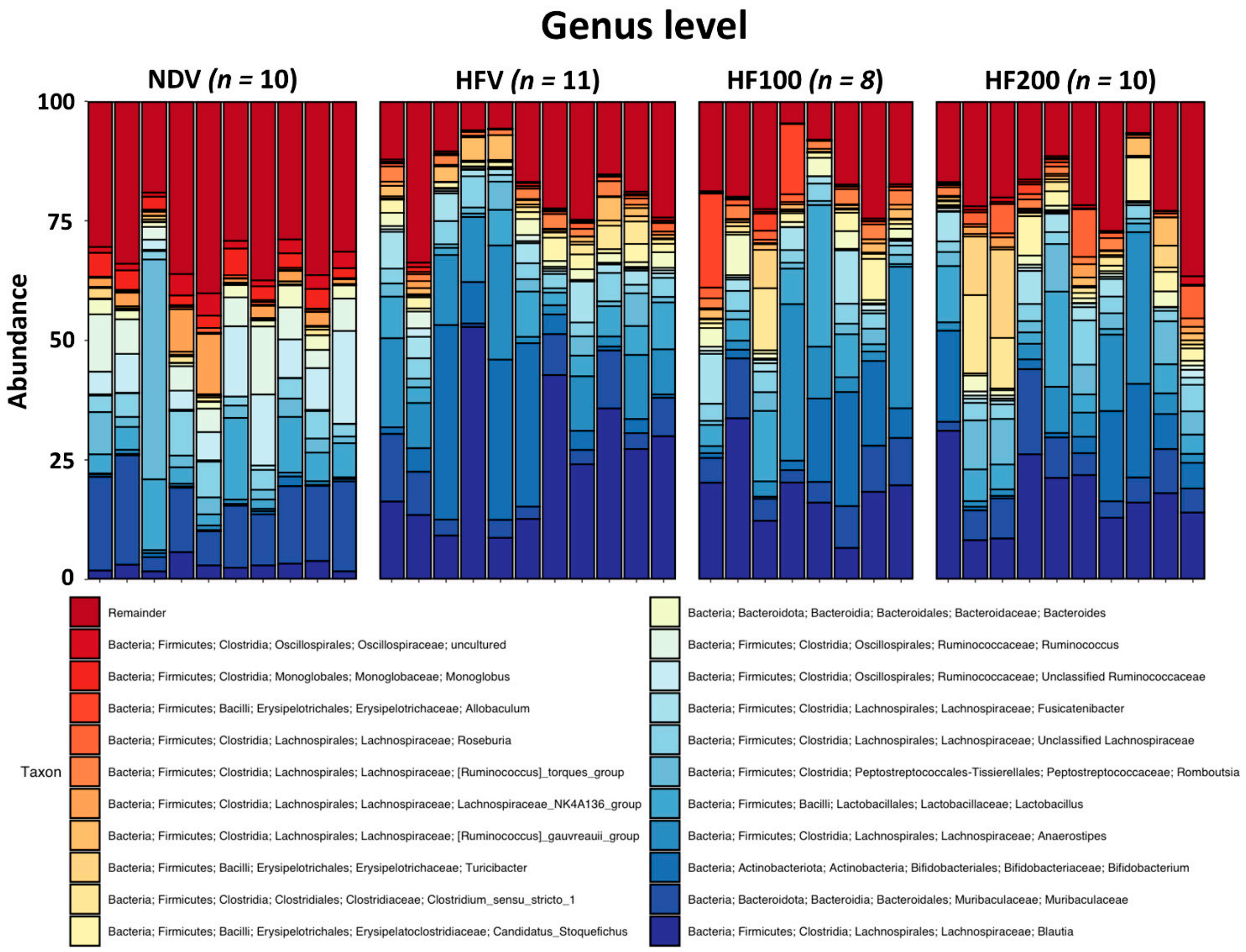

NDV: normal diet treated with vehicle

HFV: high-fat/calorie diet treated with vehicle

HF100: high-fat/calorie diet treated with 100 mg·kg^{-1}·day^{-1} of *C. terminans* extract

HF200: high-fat/calorie diet treated with 200 mg·kg^{-1}·day^{-1} of *C. terminans* extract
